# Supplementary material for: Stranger swings: Temperature-dependent upsides and downsides of a densovirus in Aedes albopictus
Source: PLoS Negl Trop Dis. 2026 Jun 8;20(6):e0014405. doi: 10.1371/journal.pntd.0014405 (PMC13245773; doi:10.1371/journal.pntd.0014405)
Supplement: S1 Table — The table presents the survival data of mosquitoes from larval to adult stages under varying temperatures and exposure to the AalDV2 virus across different blocks. The percentage of surviving mosquitoes and its 95% confidence intervals (CI) were calculated with the binconf function from the Hmisc package in R. (DOCX) [file pntd.0014405.s001.docx]

| Bloc | Temperature | Treatment | Total larvae | Dead Larvae | Dead pupae | Dead adult at emergence | Total Death | Surviving after emergence | Percentage of surviving mosquitoes | IntConf_low of the Percentage of surviving mosquitoes | IntConf_sup of the Percentage of surviving mosquitoes |
| --- | --- | --- | --- | --- | --- | --- | --- | --- | --- | --- | --- |
| 1 | 28 | C | 47 | 1 | 4 | 0 | 5 | 42 | 89.36 | 77.40 | 95.37 |
| 1 | 28 | If | 95 | 2 | 2 | 0 | 4 | 91 | 95.79 | 89.67 | 98.35 |
| 1 | 31 | C | 35 | 2 | 1 | 0 | 3 | 32 | 91.43 | 77,62 | 97.04 |
| 1 | 31 | If | 92 | 5 | 1 | 2 | 8 | 84 | 91.30 | 84.77 | 95.53 |
| 2 | 28 | C | 95 | 0 | 2 | 0 | 2 | 93 | 97.89 | 92,65 | 99.42 |
| 2 | 28 | If | 186 | 5 | 4 | 0 | 9 | 177 | 95.16 | 91.06 | 97.43 |
| 2 | 34 | C | 88 | 24 | 25 | 0 | 49 | 39 | 44.32 | 34.39 | 54.72 |
| 2 | 34 | If | 181 | 55 | 30 | 0 | 85 | 96 | 53.04 | 45.78 | 60.17 |
| 3 | 31 | C | 96 | 5 | 4 | 0 | 9 | 87 | 90.62 | 90.62 | 83.13 |
| 3 | 31 | If | 95 | 8 | 3 | 0 | 11 | 84 | 88.42 | 88.42 | 80.45 |
| 3 | 34 | C | 158 | 99 | 44 | 0 | 143 | 15 | 9.49 | 5.84 | 15.07 |
| 3 | 34 | If | 155 | 83 | 37 | 3 | 123 | 32 | 20.65 | 15.02 | 27.69 |

**S1 Table**: Survival of the mosquitoes up to emergence under different conditions. The table presents the survival data of mosquitoes from larval to adult stages under varying temperatures and exposure to the AalDV2 virus across different blocks. The percentage of surviving mosquitoes and its 95% confidence intervals (CI) were calculated with the *binconf* function from the *Hmisc* package in R.
