# Supplementary material for: Stranger swings: Temperature-dependent upsides and downsides of a densovirus in Aedes albopictus
Source: PLoS Negl Trop Dis. 2026 Jun 8;20(6):e0014405. doi: 10.1371/journal.pntd.0014405 (PMC13245773; doi:10.1371/journal.pntd.0014405)
Supplement: S2 Table — The response variable is the proportion of females at emergence, modeled using a binomial generalized linear mixed model (GLMM) with a logit link function. Degrees of freedom (df), chi-squared statistics (χ²), and p-values (Pr(>χ²)) are reported for each fixed effect. None of the tested effects (AalDV2 treatment or temperature) significantly influenced the sex ratio at emergence (p > 0.05). (DOCX) [file pntd.0014405.s002.docx]

| **Effect** | **χ^2^** | **df** | **Pr(>χ^2^)** |
| --- | --- | --- | --- |
| Intercept | 0.50 | 1 | 0.48 |
| AalDV2 - Exposure | 0.87 | 1 | 0.35 |
| Temperature | 1.12 | 2 | 0.57 |

**S2 Table**: Statistical analysis of the effect the temperature and the exposure to AalDV2 on the sex-ratio at emergence. The response variable is the proportion of females at emergence, modeled using a binomial generalized linear mixed model (GLMM) with a logit link function. Degrees of freedom (df), chi-squared statistics (χ²), and p-values (Pr(>χ²)) are reported for each fixed effect. None of the tested effects (AalDV2 treatment or temperature) significantly influenced the sex ratio at emergence (p > 0.05).
