# Supplementary material for: Stranger swings: Temperature-dependent upsides and downsides of a densovirus in Aedes albopictus
Source: PLoS Negl Trop Dis. 2026 Jun 8;20(6):e0014405. doi: 10.1371/journal.pntd.0014405 (PMC13245773; doi:10.1371/journal.pntd.0014405)
Supplement: S3 Table — This table presents the infection status of mosquitoes that survived to adulthood after exposure to the AalDV2 virus, categorized by sex and temperature. The percentage of infected mosquitoes and its 95% confidence intervals (CI) were calculated with the binconf function from the Hmisc package in R. (DOCX) [file pntd.0014405.s003.docx]

| **Sex** | **Temperature** | **Number of mosquitoes surviving emergence after exposure to AalDV2** | **Number of non-infected at adulthood** | **Percentage infected** | **IntConf_low** | **IntConf_sup** |
| --- | --- | --- | --- | --- | --- | --- |
| Male | 28 | 134 | 3 | 97.76 | 97.76 | 93.62 |
| Male | 31 | 84 | 1 | 98.81 | 98.81 | 93.56 |
| Male | 34 | 58 | 1 | 98.28 | 98.28 | 90.86 |
| Female | 28 | 134 | 5 | 96.27 | 96.27 | 91.56 |
| Female | 31 | 84 | 1 | 98.81 | 98.81 | 93.56 |
| Female | 34 | 70 | 0 | 100 | 100 | 94.8 |

**S3 Table**: **Percentage of AalDV2 infection in adult mosquitoes by sex and temperature.** This table presents the infection status of mosquitoes that survived to adulthood after exposure to the AalDV2 virus, categorized by sex and temperature. The percentage of infected mosquitoes and its 95% confidence intervals (CI) were calculated with the binconf function from the Hmisc package in R.
