# Supplementary material for: Stranger swings: Temperature-dependent upsides and downsides of a densovirus in Aedes albopictus
Source: PLoS Negl Trop Dis. 2026 Jun 8;20(6):e0014405. doi: 10.1371/journal.pntd.0014405 (PMC13245773; doi:10.1371/journal.pntd.0014405)
Supplement: Attachment — Submitted filename: Response_to_Reviewers.docx [file pntd.0014405.s006.docx]

**Journal Requirements:**

1) Please ensure that the CRediT author contributions listed for every co-author are completed accurately and in full.
At this stage, the following Authors/Authors require contributions: Christophe Boëte, Marco Perriat-Sanguinet, Anne-Sophie Gosselin-Grenet, Patrick Makoundou, Mylène Ogliastro, Mathieu Sicard, Sandra Unal, Mylène Weill, and Célestine Atyame. Please ensure that the full contributions of each author are acknowledged in the "Add/Edit/Remove Authors" section of our submission form.
The list of CRediT author contributions may be found here: https://journals.plos.org/plosntds/s/authorship#loc-author-contributions

**RESPONSE**: The contributions of every author of the manuscript have now been added in a dedicated section.

2) We ask that a manuscript source file is provided at Revision. Please upload your manuscript file as a .doc, .docx, .rtf or .tex. If you are providing a .tex file, please upload it under the item type u2018LaTeX Source Fileu2019 and leave your .pdf version as the item type u2018Manuscriptu2019.

**RESPONSE**: done

3) Please provide an Author Summary. This should appear in your manuscript between the Abstract (if applicable) and the Introduction, and should be 150-200 words long. The aim should be to make your findings accessible to a wide audience that includes both scientists and non-scientists. Sample summaries can be found on our website under Submission Guidelines:
https://journals.plos.org/plosntds/s/submission-guidelines#loc-parts-of-a-submission

**RESPONSE**: The Author Summary has now been added.

4) We do not publish any copyright or trademark symbols that usually accompany proprietary names, eg ©,  ®, or TM  (e.g. next to drug or reagent names). Therefore please remove all instances of trademark/copyright symbols throughout the text, including:
- ® on page: 5.

**RESPONSE**: The ® symbol on page 5 has been removed.

5) We have noticed that you have uploaded Supporting Information files, but you have not included a list of legends. Please add a full list of legends for your Supporting Information files after the references list.

**RESPONSE:** The full list of legends for the Supporting Information files has now been added after the reference list.

6) Please amend your detailed Financial Disclosure statement. This is published with the article. It must therefore be completed in full sentences and contain the exact wording you wish to be published.
- State the initials, alongside each funding source, of each author to receive each grant. For example: "This work was supported by the National Institutes of Health (####### to AM; ###### to CJ) and the National Science Foundation (###### to AM)."
- State what role the funders took in the study. If the funders had no role in your study, please state: "The funders had no role in study design, data collection and analysis, decision to publish, or preparation of the manuscript.".
If you did not receive any funding for this study, please simply state: u201cThe authors received no specific funding for this work.u201d
 
**RESPONSE**: done

**Reviewers' Comments:**

Reviewer's Responses to Questions

**Key Review Criteria Required for Acceptance?**


As you describe the new analyses required for acceptance, please consider the following:


**Methods**


-Are the objectives of the study clearly articulated with a clear testable hypothesis stated?

-Is the study design appropriate to address the stated objectives?

-Is the population clearly described and appropriate for the hypothesis being tested?

-Is the sample size sufficient to ensure adequate power to address the hypothesis being tested?

-Were correct statistical analysis used to support conclusions?

-Are there concerns about ethical or regulatory requirements being met?

Reviewer #1: I am not familiar with fluctuation asymmetry and I feel the authors could do a better job describing this phenotypic measurement and what it is supposed to actually represent. Basically, provide more details on this measurement.

RESPONSE:

We thank Reviewer #1 for this suggestion to better explain fluctuating asymmetry. We have expanded the description of fluctuating asymmetry (FA) in the Methods section. FA measures random, non-directional deviations from perfect bilateral symmetry in paired morphological traits here, wing length. Contrary to directional asymmetry, FA arises from the inability of developmental processes to buffer stochastic perturbations and is often used as an indicator of developmental stability: individuals under greater environmental or physiological stress tend to exhibit higher FA values. This has been the case for wing length in insects but also in birds for example. This explanation, along with the relevant reference (Palmer & Strobeck 1986), has been added to the Methods section.

Reviewer #2: -I think the entire design needs to be replicated on a different generation of mosquitoes. Fitness and virus effects can be variable and if you see them on one single cohort of mosquitoes there could have been something distinct going on (environmental or biotic effects). Blocking internally does not fix this issue. Repeat to see if it happens again.
-Sample sizes (or at least minimums in treatments and blocks) should be given in the methods. There are A LOT of statistics being done here and I am concerned the depth of data do not warrant this level of analysis.
-Is the gene expression simply the ratio to a host gene or is it the delta delta CT calculation? Include reference and proper description.
-Same for the wing length methods - how and reference

RESPONSE: We thank Reviewer #2 for the methodological comments. Below we address each point.

(1) Replication: The experiment was conducted in three independent blocks run at different time points using mosquitoes from successive generations of our colony, providing genuine temporal and generational replication. Each temperature × treatment combination was represented in two independent blocks and the protective effect of AalDV2 at 34°C was observed consistently in both replicates at that temperature, in the same direction and of comparable magnitude. We agree that this differs from replication using a fully independent colony, and we now acknowledge this explicitly in the Discussion as an avenue for future confirmation. However, we note that the consistency of the effect across independent blocks, combined with its statistical robustness, provides substantial support for the biological reality of our findings.

Sample sizes: The detailed per-group sample sizes are already provided in Supplementary Table S1, which reports total numbers of larvae, deaths at each developmental stage, and survivors for every combination bloc × temperature × treatment. Regarding the depth of statistical analysis, each test addresses a distinct biological question (survival, developmental timing, body size, developmental stability, and viral load), and model selection was guided by the distributional properties of each response variable with appropriate robustness checks. We are therefore confident that the analyses are warranted by the data.

(3) Gene expression/viral load: Here is a clarification requested by the reviewer. Viral load was quantified as the ratio of AalDV2 genome copies to the host actin single-copy locus, using relative quantification of DNA. It reflects the relative number of viral genome copies per mosquito genome equivalent. The Methods section now explicitly states this point.
(4) Wing length: We have expanded the wing measurement description. Wing length was measured from the axillary incision to the apical margin under a stereomicroscope using a calibrated precision scale, following standard methodology for *Aedes* mosquitoes (Armbruster & Hutchinson 2002), a reference already cited in the manuscript for the wing size–fecundity relationship.

Reviewer #3: (No Response)

RESPONSE: n/a

Reviewer #4: -Are the objectives of the study clearly articulated with a clear testable hypothesis stated?
Ans: Yes. The objective is clearly stated, however the testable hypothesis is not explicitly formulated.
-Is the study design appropriate to address the stated objectives?
Ans: Yes
-Is the population clearly described and appropriate for the hypothesis being tested?
Ans: Yes the study population (Ades albopictus) is appropriate for addressing the research question.
-Is the sample size sufficient to ensure adequate power to address the hypothesis being tested?
Ans: The manuscript does not provide sufficient details to assess whether the sample size was adequate. The authors should report the number of individuals per treatment group and report how sample size was determined, preferably with power analysis or justification.
-Were correct statistical analysis used to support conclusions?
Ans; Yes the statistical analyses used appear appropriate for type of data presented.
-Are there concerns about ethical or regulatory requirements being met?
The study involves standard laboratory work with mosquito larvae and cell cultures. However, the author may include a brief statement confirming compliance with institutional guidelines if required by journal.

RESPONSE: We thank Reviewer #4 for the evaluation. Following these comments, we have:

(1) added a hypothesis statement at the end of the Introduction, stating that we hypothesized AalDV2 infection would impose fitness costs across all temperatures and that higher temperatures would exacerbate these negative effects;

(2) added a sentence in the Methods directing readers to Supplementary Table S1. This latter one already provides total numbers of individuals per bloc, temperature, and treatment combination, as well as survival outcomes at each developmental stage;

(3) included a brief compliance statement in the Methods confirming that all procedures followed institutional biosafety regulations at ISEM, Université de Montpellier, and that no specific ethical approval is required under French institutional guidelines for invertebrate laboratory research.

**Results**


-Does the analysis presented match the analysis plan?

-Are the results clearly and completely presented?

-Are the figures (Tables, Images) of sufficient quality for clarity?

Reviewer #1: Yes, the authors had a very well articulated plan and did a very nice job in executing it. The accompanying statistical analyses were great and the figures and tables were clear.

RESPONSE:  We thank Reviewer #1 for the very positive assessment of our study design, statistical analyses, and presentation of results.

Reviewer #2: -Three points (temperatures) is just barely a line. So I would probably refrain from talking about 'non linear' effects and instead speak about threshold or unique differences at the higher temperature.
-If the stats are largely in parentheses in the results you do not also need a table. Quote in the text and put the full table in the supp if there are a few lines you don't mention. It is redundant.
-Sample sizes should be on all figures or in legends. Hard to evaluate the impact of the work. Were there only 6 mosquitoes measured in each treatment for wing length for example?

RESPONSE:

We have been taking into account the observations made by Reviewer #2 and have addressed them as follows:

(1) we have replaced the term 'non-linear' throughout the manuscript and refer now to a threshold effect and temperature-specific differences. We agree that three temperature points can be considered insufficient to formally characterize a non-linear response curve. We have therefore replaced 'non-linear' with 'threshold-like' throughout, which more accurately reflects what our data demonstrate: a qualitative shift in both survival and virus-host dynamics above 31°C, without claiming to describe the full shape of the response.
(2) We respectfully disagree with this suggestion. Key statistics are indeed reported in the Results text for readability and the tables present the complete model output. This includes all fixed effects, interactions, and degrees of freedom in a format that allows readers to fully evaluate the analyses without hunting through paragraphs of text. This is standard practice for papers reporting linear and generalized linear mixed models, where transparency of the full model structure is important. We have therefore retained the tables in the main text.
(3) Sample sizes have been added to all figure legends. To clarify, the apparent small number of data points visible in some figures does not reflect the actual sample sizes, which are quite substantial as indicated in the supplementary tables and now explicitly stated in the relevant figure legends.

Reviewer #3: (No Response)

RESPONSE:  n/a

Reviewer #4: -Does the analysis presented match the analysis plan?
Ans: The analysis presented are consistent with analysis plan in method section. No major discrepancies are apparent.
-Are the results clearly and completely presented?
Ans: Yes
-Are the figures (Tables, Images) of sufficient quality for clarity?
Ans. Yes

RESPONSE:  We thank Reviewer #4 for the positive overall assessment of our manuscript.

**Conclusions**


-Are the conclusions supported by the data presented?

-Are the limitations of analysis clearly described?

-Do the authors discuss how these data can be helpful to advance our understanding of the topic under study?

-Is public health relevance addressed?

Reviewer #1: The conclusions were well articulated and are consistent with their findings. The authors do a nice job discussing their findings in a larger context and the importance of examining new and emerging biocontrol under more complex environmentally realistic conditions.

RESPONSE:  We thank Reviewer #1 for the positive evaluations of our conclusion and discussion.

Reviewer #2: (No Response)

RESPONSE: n/a

Reviewer #3: (No Response)

RESPONSE: n/a

Reviewer #4: -Are the conclusions supported by the data presented?
Ans: Yes
-Are the limitations of analysis clearly described?
Ans: The limitation of study are not explicitly described but some aspects are indirectly suggested. The authors should clearly state the main limitations of the study.
-Do the authors discuss how these data can be helpful to advance our understanding of the topic under study?
Ans: Yes
-Is public health relevance addressed?
Ans: Yes

RESPONSE:

We thank Reviewer #4 for the overall positive assessment. Regarding the suggestion regarding limitations, we have added a dedicated paragraph in the Discussion section in order to better contextualizing the scope of the present findings (the restriction to aquatic stages, the use of a single viral dose and colony, the choice of constant over fluctuating temperatures, and the replication structure of the experiment). We also provide direction for future investigation.

**Editorial and Data Presentation Modifications?**


Use this section for editorial suggestions as well as relatively minor modifications of existing data that would enhance clarity. If the only modifications needed are minor and/or editorial, you may wish to recommend “Minor Revision” or “Accept”.

Reviewer #1: Accetpt

RESPONSE:  We thank Reviewer #1 for the evaluation of the manuscript and the recommendation for publication in PLoS NTD.

Reviewer #2: Some paragraph structure in the intro would help the reader.

'a number of responses have been brought' awkward

'Fluctuating' not 'fluctuation' asymmetry

'Actin' not 'actine'

RESPONSE:  We thank Reviewer #2 and the corrections have now been made.

Reviewer #3: (No Response)

RESPONSE:  n/a

Reviewer #4: The manuscript is generally well written and presents relevant findings; however, several editorial and clarity improvements are recommended. Language and grammar should be carefully revised throughout to improve readability and flow. Some sections, particularly in methods would benefits from clearer and more concise descriptions to improve readability and reproducibility. Overall, these are minor editorial changes.

RESPONSE:  We thank Reviewer #4 for the overall positive assessment of the manuscript and editorial changes have been made to improve grammar, readability and flow.

**Summary and General Comments**


Use this section to provide overall comments, discuss strengths/weaknesses of the study, novelty, significance, general execution and scholarship. You may also include additional comments for the author, including concerns about dual publication, research ethics, or publication ethics. If requesting major revision, please articulate the new experiments that are needed.

Reviewer #1: Overall, this was a nice study evaluating the impacts of densovirus infection on the life history traits of Ae. albopictus under different temperatures. They measured a number of traits and performed the appropriate statistical analysis. This is a well written manuscript that shines light on the importance of testing biocontrol strategies under changing environmental conditions.

RESPONSE:  Again, we thank Reviewer #1 for the overall positive evaluation of the manuscript.

Reviewer #2: This paper explores the interaction between densovirus and temperature in several measures of Ae. albopictus fitness. Densovirus is being explored for vector control and this examines some basic biology.
The manuscript is in some places – under written and in other places over written.

Authors should include line numbers on submissions to assist reviewers in providing feedback.

RESPONSE:  We thank Reviewer #2 for the feedback. We hope the rewriting of some section are now fulfilling the expectations. Line numbers have been added.

Reviewer #3: In the Manuscript entitled "Stranger Swings: Temperature-Dependent Upsides and Downsides of a Densovirus in Aedes albopictus" by Boëte C al., the Authors investigate the effects of exposure to the densovirus AalDV2 (an entopathogenic virus), combined with different temperatures (28°C, 31°C, and 34°C) on the survival, development time, wing size and symmetry of Aedes albopictus. The results of this work confirm previous studies showing negative impacts of AalDV2 on mosquito life cycle characteristics, although, surprisingly, they highlight an unexpected beneficial protective effect of this densovirus on Ae. albopictus at 34 °C. These findings call into question the role of this virus as a potential vector control tool, underlining the need to consider temperature-dependent effects when evaluating biological control agents, especially in light of climate change.
I think the article is well written, logically ordered and the conclusions appropriate. It's unfortunate that so little attention was paid to the final version of the work, especially since it greatly complicates editing. Please, always include the page and line counts and review the bibliography more carefully in the text.
In any case, I consider it suitable for PLOS Neglected Tropical Diseases, after a minor revision.
•The species name is often reported in the text as A. albopictus or Ae. albopictus. Please standardize in the latter manner.
•In the Methods section, the species name Aedes aegypti is reported for the first time, so it should be written in full.
•Buchatsky 1989b is cited, but it is not listed in the references.
•Many citations are missing: e.g., Gorovits et al., 2022; Xu et al., 2008, 2014; Westwood et al., 2013; Selbach et al. 2020, and many others.
•Please replace Altinli et al. 2020 with Altinli et al. 2021.

RESPONSE:

We appreciate the careful reading by Reviewer #3, the positive overall assessment and the recommendation for publication in PLoS NTD after a minor revision. We apologize for the formatting mistakes in the initial submission and have ensured that page and line numbers are included in this revised version. We have addressed all specific points raised: (1) the species abbreviation has been standardized to Ae. albopictus throughout; (2) Aedes aegypti is now written in full at first mention in the Methods; (3) Buchatsky 1989 (Diseases of Aquatic Organisms, 6:145–150) has been added to the reference list; (4) all missing references cited in the text (Gorovits et al. 2022, Xu et al. 2008 and 2014,…) have now been added to the bibliography; (5) Altinli et al. 2020 has been replaced by Altinli et al. 2021.

Reviewer #4: This study addresses a relevant topic and provides insight into the interaction between temperature and AalDV2 infection in Ades albopictus. The experimental approach is appropriate and the findings are interesting. However, clarity should be improved, minor editorial revision are also needed. Overall, the manuscript is suitable for publication after minor revision.
Confidential Comments to Editor
The manuscript is sound and presents interesting results. Revisions are mainly editorial. I recommend minor revision.

RESPONSE:  We thank Reviewer #4 for the positive overall assessment and the recommendation for publication in PLoS NTD after a minor revision. All editorial and clarity improvements suggested have been implemented.

[NOTE: If reviewer comments were submitted as an attachment file, they will be attached to this email and accessible via the submission site. Please log into your account, locate the manuscript record, and check for the action link "View Attachments". If this link does not appear, there are no attachment files.]

**Figure resubmission:**

While revising your submission, we strongly recommend that you use PLOS’s NAAS tool (https://ngplosjournals.pagemajik.ai/artanalysis) to test your figure files. NAAS can convert your figure files to the TIFF file type and meet basic requirements (such as print size, resolution), or provide you with a report on issues that do not meet our requirements and that NAAS cannot fix.

After uploading your figures to PLOS’s NAAS tool - https://ngplosjournals.pagemajik.ai/artanalysis, NAAS will process the files provided and display the results in the "Uploaded Files" section of the page as the processing is complete. If the uploaded figures meet our requirements (or NAAS is able to fix the files to meet our requirements), the figure will be marked as "fixed" above. If NAAS is unable to fix the files, a red "failed" label will appear above. When NAAS has confirmed that the figure files meet our requirements, please download the file via the download option, and include these NAAS processed figure files when submitting your revised manuscript.

**Reproducibility:**

To enhance the reproducibility of your results, we recommend that authors of applicable studies deposit laboratory protocols in protocols.io, where a protocol can be assigned its own identifier (DOI) such that it can be cited independently in the future. Additionally, PLOS ONE offers an option to publish peer-reviewed clinical study protocols. Read more information on sharing protocols at https://plos.org/protocols?utm_medium=editorial-email&utm_source=authorletters&utm_campaign=protocols
