# Supplementary material for: Stranger swings: Temperature-dependent upsides and downsides of a densovirus in Aedes albopictus
Source: PLoS Negl Trop Dis. 2026 Jun 8;20(6):e0014405. doi: 10.1371/journal.pntd.0014405 (PMC13245773; doi:10.1371/journal.pntd.0014405)
Supplement: Peer review history

# Peer review: Decision Letter 0

**DOI:** 10.1371/journal.pntd.0014405.r001

**Authors:** Audrey Lenhart, Ran Wang

6 May 2026

PNTD-D-26-00726

Stranger Swings: Temperature-Dependent Upsides and Downsides of a Densovirus in Aedes albopictus

PLOS Neglected Tropical Diseases

Dear Dr. Boëte,

Thank you for submitting your manuscript to PLOS Neglected Tropical Diseases. After careful consideration, we feel that it has merit but does not fully meet PLOS Neglected Tropical Diseases's publication criteria as it currently stands. Therefore, we invite you to submit a revised version of the manuscript that addresses the points raised during the review process.

Please submit your revised manuscript within by Jul 05 2026 11:59PM. If you will need more time than this to complete your revisions, please reply to this message or contact the journal office at plosntds@plos.org. When you're ready to submit your revision, log on to https://www.editorialmanager.com/pntd/ and select the 'Submissions Needing Revision' folder to locate your manuscript file.

Please include the following items when submitting your revised manuscript:

* A letter that responds to each point raised by the editor and reviewer(s). You should upload this letter as a separate file labeled 'Response to Reviewers'. This file does not need to include responses to any formatting updates and technical items listed in the 'Journal Requirements' section below.

* A marked-up copy of your manuscript that highlights changes made to the original version. You should upload this as a separate file labeled 'Revised Manuscript with Track Changes'.

* An unmarked version of your revised paper without tracked changes. You should upload this as a separate file labeled 'Manuscript'.

If you would like to make changes to your financial disclosure, competing interests statement, or data availability statement, please make these updates within the submission form at the time of resubmission. Guidelines for resubmitting your figure files are available below the reviewer comments at the end of this letter.

As the corresponding author, your ORCID iD is verified in the submission system and will appear in the published article. PLOS supports the use of ORCID, and we encourage all coauthors to register for an ORCID iD and use it as well. Please encourage your coauthors to verify their ORCID iD within the submission system before final acceptance, as unverified ORCID iDs will not appear in the published article. Only the individual author can complete the verification step; PLOS staff cannot verify ORCID iDs on behalf of authors.

We look forward to receiving your revised manuscript.

Kind regards,

Ran Wang, M.D.

Academic Editor

PLOS Neglected Tropical Diseases

Audrey Lenhart

Section Editor

PLOS Neglected Tropical Diseases

Shaden Kamhawi

co-Editor-in-Chief

PLOS Neglected Tropical Diseases

orcid.org/0000-0003-4304-636XX

Paul Brindley

co-Editor-in-Chief

PLOS Neglected Tropical Diseases

orcid.org/0000-0003-1765-0002

**Journal Requirements:**

1) Please ensure that the CRediT author contributions listed for every co-author are completed accurately and in full.

At this stage, the following Authors/Authors require contributions: Christophe Boëte, Marco Perriat-Sanguinet, Anne-Sophie Gosselin-Grenet, Patrick Makoundou, Mylène Ogliastro, Mathieu Sicard, Sandra Unal, Mylène Weill, and Célestine Atyame. Please ensure that the full contributions of each author are acknowledged in the "Add/Edit/Remove Authors" section of our submission form.

The list of CRediT author contributions may be found here: https://journals.plos.org/plosntds/s/authorship#loc-author-contributions

2) We ask that a manuscript source file is provided at Revision. Please upload your manuscript file as a .doc, .docx, .rtf or .tex. If you are providing a .tex file, please upload it under the item type u2018LaTeX Source Fileu2019 and leave your .pdf version as the item type u2018Manuscriptu2019.

3) Please provide an Author Summary. This should appear in your manuscript between the Abstract (if applicable) and the Introduction, and should be 150-200 words long. The aim should be to make your findings accessible to a wide audience that includes both scientists and non-scientists. Sample summaries can be found on our website under Submission Guidelines:

https://journals.plos.org/plosntds/s/submission-guidelines#loc-parts-of-a-submission

4) We do not publish any copyright or trademark symbols that usually accompany proprietary names, eg ©,  ®, or TM  (e.g. next to drug or reagent names). Therefore please remove all instances of trademark/copyright symbols throughout the text, including:

- ® on page: 5.

5) We have noticed that you have uploaded Supporting Information files, but you have not included a list of legends. Please add a full list of legends for your Supporting Information files after the references list.

6) Please amend your detailed Financial Disclosure statement. This is published with the article. It must therefore be completed in full sentences and contain the exact wording you wish to be published.

- State the initials, alongside each funding source, of each author to receive each grant. For example: "This work was supported by the National Institutes of Health (####### to AM; ###### to CJ) and the National Science Foundation (###### to AM)."

- State what role the funders took in the study. If the funders had no role in your study, please state: "The funders had no role in study design, data collection and analysis, decision to publish, or preparation of the manuscript.".

If you did not receive any funding for this study, please simply state: u201cThe authors received no specific funding for this work.u201d

**Reviewers' Comments:**

Reviewer's Responses to Questions

**Key Review Criteria Required for Acceptance?**

As you describe the new analyses required for acceptance, please consider the following:

**Methods**

-Are the objectives of the study clearly articulated with a clear testable hypothesis stated?

-Is the study design appropriate to address the stated objectives?

-Is the population clearly described and appropriate for the hypothesis being tested?

-Is the sample size sufficient to ensure adequate power to address the hypothesis being tested?

-Were correct statistical analysis used to support conclusions?

-Are there concerns about ethical or regulatory requirements being met?

Reviewer #1: I am not familiar with fluctuation asymmetry and I feel the authors could do a better job describing this phenotypic measurement and what it is supposed to actually represent. Basically, provide more details on this measurement.

Reviewer #2: -I think the entire design needs to be replicated on a different generation of mosquitoes. Fitness and virus effects can be variable and if you see them on one single cohort of mosquitoes there could have been something distinct going on (environmental or biotic effects). Blocking internally does not fix this issue. Repeat to see if it happens again.

-Sample sizes (or at least minimums in treatments and blocks) should be given in the methods. There are A LOT of statistics being done here and I am concerned the depth of data do not warrant this level of analysis.

-Is the gene expression simply the ratio to a host gene or is it the delta delta CT calculation? Include reference and proper description.

-Same for the wing length methods - how and reference

Reviewer #3: (No Response)

Reviewer #4: -Are the objectives of the study clearly articulated with a clear testable hypothesis stated?

Ans: Yes. The objective is clearly stated, however the testable hypothesis is not explicitly formulated.

-Is the study design appropriate to address the stated objectives?

Ans: Yes

-Is the population clearly described and appropriate for the hypothesis being tested?

Ans: Yes the study population (Ades albopictus) is appropriate for addressing the research question.

-Is the sample size sufficient to ensure adequate power to address the hypothesis being tested?

Ans: The manuscript does not provide sufficient details to assess whether the sample size was adequate. The authors should report the number of individuals per treatment group and report how sample size was determined, preferably with power analysis or justification.

-Were correct statistical analysis used to support conclusions?

Ans; Yes the statistical analyses used appear appropriate for type of data presented.

-Are there concerns about ethical or regulatory requirements being met?

The study involves standard laboratory work with mosquito larvae and cell cultures. However, the author may include a brief statement confirming compliance with institutional guidelines if required by journal.

**********

**Results**

-Does the analysis presented match the analysis plan?

-Are the results clearly and completely presented?

-Are the figures (Tables, Images) of sufficient quality for clarity?

Reviewer #1: Yes, the authors had a very well articulated plan and did a very nice job in executing it. The accompanying statistical analyses were great and the figures and tables were clear.

Reviewer #2: -Three points (temperatures) is just barely a line. So I would probably refrain from talking about 'non linear' effects and instead speak about threshold or unique differences at the higher temperature.

-If the stats are largely in parentheses in the results you do not also need a table. Quote in the text and put the full table in the supp if there are a few lines you don't mention. It is redundant.

-Sample sizes should be on all figures or in legends. Hard to evaluate the impact of the work. Were there only 6 mosquitoes measured in each treatment for wing length for example?

Reviewer #3: (No Response)

Reviewer #4: -Does the analysis presented match the analysis plan?

Ans: The analysis presented are consistent with analysis plan in method section. No major discrepancies are apparent.

-Are the results clearly and completely presented?

Ans: Yes

-Are the figures (Tables, Images) of sufficient quality for clarity?

Ans. Yes

**********

**Conclusions**

-Are the conclusions supported by the data presented?

-Are the limitations of analysis clearly described?

-Do the authors discuss how these data can be helpful to advance our understanding of the topic under study?

-Is public health relevance addressed?

Reviewer #1: The conclusions were well articulated and are consistent with their findings. The authors do a nice job discussing their findings in a larger context and the importance of examining new and emerging biocontrol under more complex environmentally realistic conditions.

Reviewer #2: (No Response)

Reviewer #3: (No Response)

Reviewer #4: -Are the conclusions supported by the data presented?

Ans: Yes

-Are the limitations of analysis clearly described?

Ans: The limitation of study are not explicitly described but some aspects are indirectly suggested. The authors should clearly state the main limitations of the study.

-Do the authors discuss how these data can be helpful to advance our understanding of the topic under study?

Ans: Yes

-Is public health relevance addressed?

Ans: Yes

**********

**Editorial and Data Presentation Modifications?**

Use this section for editorial suggestions as well as relatively minor modifications of existing data that would enhance clarity. If the only modifications needed are minor and/or editorial, you may wish to recommend “Minor Revision” or “Accept”.

Reviewer #1: Accetpt

Reviewer #2: Some paragraph structure in the intro would help the reader.

'a number of responses have been brought' awkward

'Fluctuating' not 'fluctuation' asymmetry

'Actin' not 'actine'

Reviewer #3: (No Response)

Reviewer #4: The manuscript is generally well written and presents relevant findings; however, several editorial and clarity improvements are recommended. Language and grammar should be carefully revised throughout to improve readability and flow. Some sections, particularly in methods would benefits from clearer and more concise descriptions to improve readability and reproducibility. Overall, these are minor editorial changes.

**********

**Summary and General Comments**

Use this section to provide overall comments, discuss strengths/weaknesses of the study, novelty, significance, general execution and scholarship. You may also include additional comments for the author, including concerns about dual publication, research ethics, or publication ethics. If requesting major revision, please articulate the new experiments that are needed.

Reviewer #1: Overall, this was a nice study evaluating the impacts of densovirus infection on the life history traits of Ae. albopictus under different temperatures. They measured a number of traits and performed the appropriate statistical analysis. This is a well written manuscript that shines light on the importance of testing biocontrol strategies under changing environmental conditions.

Reviewer #2: This paper explores the interaction between densovirus and temperature in several measures of Ae. albopictus fitness. Densovirus is being explored for vector control and this examines some basic biology.

The manuscript is in some places – under written and in other places over written.

Authors should include line numbers on submissions to assist reviewers in providing feedback.

Reviewer #3: In the Manuscript entitled "Stranger Swings: Temperature-Dependent Upsides and Downsides of a Densovirus in Aedes albopictus" by Boëte C al., the Authors investigate the effects of exposure to the densovirus AalDV2 (an entopathogenic virus), combined with different temperatures (28°C, 31°C, and 34°C) on the survival, development time, wing size and symmetry of Aedes albopictus. The results of this work confirm previous studies showing negative impacts of AalDV2 on mosquito life cycle characteristics, although, surprisingly, they highlight an unexpected beneficial protective effect of this densovirus on Ae. albopictus at 34 °C. These findings call into question the role of this virus as a potential vector control tool, underlining the need to consider temperature-dependent effects when evaluating biological control agents, especially in light of climate change.

I think the article is well written, logically ordered and the conclusions appropriate. It's unfortunate that so little attention was paid to the final version of the work, especially since it greatly complicates editing. Please, always include the page and line counts and review the bibliography more carefully in the text.

In any case, I consider it suitable for PLOS Neglected Tropical Diseases, after a minor revision.

•The species name is often reported in the text as A. albopictus or Ae. albopictus. Please standardize in the latter manner.

•In the Methods section, the species name Aedes aegypti is reported for the first time, so it should be written in full.

•Buchatsky 1989b is cited, but it is not listed in the references.

•Many citations are missing: e.g., Gorovits et al., 2022; Xu et al., 2008, 2014; Westwood et al., 2013; Selbach et al. 2020, and many others.

•Please replace Altinli et al. 2020 with Altinli et al. 2021.

Reviewer #4: This study addresses a relevant topic and provides insight into the interaction between temperature and AalDV2 infection in Ades albopictus. The experimental approach is appropriate and the findings are interesting. However, clarity should be improved, minor editorial revision are also needed. Overall, the manuscript is suitable for publication after minor revision.

Confidential Comments to Editor

The manuscript is sound and presents interesting results. Revisions are mainly editorial. I recommend minor revision.

[NOTE: If reviewer comments were submitted as an attachment file, they will be attached to this email and accessible via the submission site. Please log into your account, locate the manuscript record, and check for the action link "View Attachments". If this link does not appear, there are no attachment files.]

**Figure resubmission:** While revising your submission, we strongly recommend that you use PLOS’s NAAS tool (https://ngplosjournals.pagemajik.ai/artanalysis) to test your figure files. NAAS can convert your figure files to the TIFF file type and meet basic requirements (such as print size, resolution), or provide you with a report on issues that do not meet our requirements and that NAAS cannot fix.

After uploading your figures to PLOS’s NAAS tool - https://ngplosjournals.pagemajik.ai/artanalysis, NAAS will process the files provided and display the results in the "Uploaded Files" section of the page as the processing is complete. If the uploaded figures meet our requirements (or NAAS is able to fix the files to meet our requirements), the figure will be marked as "fixed" above. If NAAS is unable to fix the files, a red "failed" label will appear above. When NAAS has confirmed that the figure files meet our requirements, please download the file via the download option, and include these NAAS processed figure files when submitting your revised manuscript.

**Reproducibility:**

To enhance the reproducibility of your results, we recommend that authors of applicable studies deposit laboratory protocols in protocols.io, where a protocol can be assigned its own identifier (DOI) such that it can be cited independently in the future. Additionally, PLOS ONE offers an option to publish peer-reviewed clinical study protocols. Read more information on sharing protocols at https://plos.org/protocols?utm_medium=editorial-email&utm_source=authorletters&utm_campaign=protocols

Attachment Submitted filename: PNTD-D-26-00726-lv.pdf

# Author comment: Author response to Decision Letter 1

**DOI:** 10.1371/journal.pntd.0014405.r002

15 May 2026

Attachment Submitted filename: Response_to_Reviewers.docx

# Peer review: Decision Letter 1

**DOI:** 10.1371/journal.pntd.0014405.r003

**Authors:** Audrey Lenhart, Ran Wang

22 May 2026

Dear Boëte,

We are pleased to inform you that your manuscript 'Stranger Swings: Temperature-Dependent Upsides and Downsides of a Densovirus in Aedes albopictus' has been provisionally accepted for publication in PLOS Neglected Tropical Diseases.

Before your manuscript can be formally accepted you will need to complete some formatting changes, which you will receive in a follow up email. A member of our team will be in touch with a set of requests.

Please note that your manuscript will not be scheduled for publication until you have made the required changes, so a swift response is appreciated.

IMPORTANT: The editorial review process is now complete. PLOS will only permit corrections to spelling, formatting or significant scientific errors from this point onwards. Requests for major changes, or any which affect the scientific understanding of your work, will cause delays to the publication date of your manuscript.

Should you, your institution's press office or the journal office choose to press release your paper, you will automatically be opted out of early publication. We ask that you notify us now if you or your institution is planning to press release the article. All press must be co-ordinated with PLOS.

Thank you again for supporting Open Access publishing; we are looking forward to publishing your work in PLOS Neglected Tropical Diseases.

Best regards,

Ran Wang, M.D.

Academic Editor

PLOS Neglected Tropical Diseases

Audrey Lenhart

Section Editor

PLOS Neglected Tropical Diseases

Shaden Kamhawi

co-Editor-in-Chief

PLOS Neglected Tropical Diseases

orcid.org/0000-0003-4304-636XX

Paul Brindley

co-Editor-in-Chief

PLOS Neglected Tropical Diseases

orcid.org/0000-0003-1765-0002

***********************************************************

Reviewer's Responses to Questions

**Key Review Criteria Required for Acceptance?**

As you describe the new analyses required for acceptance, please consider the following:

**Methods**

-Are the objectives of the study clearly articulated with a clear testable hypothesis stated?

-Is the study design appropriate to address the stated objectives?

-Is the population clearly described and appropriate for the hypothesis being tested?

-Is the sample size sufficient to ensure adequate power to address the hypothesis being tested?

-Were correct statistical analysis used to support conclusions?

-Are there concerns about ethical or regulatory requirements being met?

Reviewer #1: (No Response)

Reviewer #2: (No Response)

Reviewer #3: (No Response)

**********

**Results**

-Does the analysis presented match the analysis plan?

-Are the results clearly and completely presented?

-Are the figures (Tables, Images) of sufficient quality for clarity?

Reviewer #1: (No Response)

Reviewer #2: (No Response)

Reviewer #3: (No Response)

**********

**Conclusions**

-Are the conclusions supported by the data presented?

-Are the limitations of analysis clearly described?

-Do the authors discuss how these data can be helpful to advance our understanding of the topic under study?

-Is public health relevance addressed?

Reviewer #1: (No Response)

Reviewer #2: (No Response)

Reviewer #3: (No Response)

**********

**Editorial and Data Presentation Modifications?**

Use this section for editorial suggestions as well as relatively minor modifications of existing data that would enhance clarity. If the only modifications needed are minor and/or editorial, you may wish to recommend “Minor Revision” or “Accept”.

Reviewer #1: (No Response)

Reviewer #2: (No Response)

Reviewer #3: (No Response)

**********

**Summary and General Comments**

Use this section to provide overall comments, discuss strengths/weaknesses of the study, novelty, significance, general execution and scholarship. You may also include additional comments for the author, including concerns about dual publication, research ethics, or publication ethics. If requesting major revision, please articulate the new experiments that are needed.

Reviewer #1: The authors addressed all of my concerns and those of the other reviewers.

Reviewer #2: (No Response)

Reviewer #3: (No Response)

**********

PLOS authors have the option to publish the peer review history of their article (what does this mean?). If published, this will include your full peer review and any attached files.

If you choose “no”, your identity will remain anonymous but your review may still be made public.

**Do you want your identity to be public for this peer review?** For information about this choice, including consent withdrawal, please see our Privacy Policy.

Reviewer #1: **Yes:**Doug E Brackney

Reviewer #2: No

Reviewer #3: No

# Editor report: Acceptance letter

**DOI:** 10.1371/journal.pntd.0014405.r004

**Authors:** Audrey Lenhart, Ran Wang

Dear Boëte,

We are delighted to inform you that your manuscript, "Stranger Swings: Temperature-Dependent Upsides and Downsides of a Densovirus in Aedes albopictus," has been formally accepted for publication in PLOS Neglected Tropical Diseases.

We have now passed your article onto the PLOS Production Department who will complete the rest of the publication process. All authors will receive a confirmation email upon publication.

The corresponding author will soon be receiving a typeset proof for review, to ensure errors have not been introduced during production. Please review the PDF proof of your manuscript carefully, as this is the last chance to correct any scientific or type-setting errors. Please note that major changes, or those which affect the scientific understanding of the work, will likely cause delays to the publication date of your manuscript. Note: Proofs for Front Matter articles (Editorial, Viewpoint, Symposium, Review, etc...) are generated on a different schedule and may not be made available as quickly.

Soon after your final files are uploaded, the early version of your manuscript will be published online unless you opted out of this process. The date of the early version will be your article's publication date. The final article will be published to the same URL, and all versions of the paper will be accessible to readers.

For Research Articles, you will receive an invoice from PLOS for your publication fee after your manuscript has reached the completed accept phase. If you receive an email requesting payment before acceptance or for any other service, this may be a phishing scheme. Learn how to identify phishing emails and protect your accounts at https://explore.plos.org/phishing.

Thank you again for supporting open-access publishing; we are looking forward to publishing your work in PLOS Neglected Tropical Diseases.

Best regards,

Shaden Kamhawi

co-Editor-in-Chief

PLOS Neglected Tropical Diseases

Paul Brindley

co-Editor-in-Chief

PLOS Neglected Tropical Diseases
